# Twenty-year trajectories of morbidity in individuals with and without osteoarthritis

**DOI:** 10.1136/rmdopen-2024-004164

**Published:** 2024-07-02

**Authors:** Andrea Dell'Isola, Filippo Recenti, Martin Englund, Ali Kiadaliri

**Affiliations:** 1 Clinical Epidemiology Unit, Orthopedics, Department of Clinical Sciences Lund, Lund University, Lund, Sweden; 2 Department of Neurosciences, Rehabilitation, Ophthalmology, Genetics, Maternal and Child Health, University of Genoa, Savona, Italy

**Keywords:** Osteoarthritis, Sweden, Hypertension

## Abstract

**Objectives:**

To identify multimorbidity trajectories over 20 years among incident osteoarthritis (OA) individuals and OA-free matched references.

**Methods:**

Cohort study using prospectively collected healthcare data from the Skåne region, Sweden (~1.4 million residents). We extracted diagnoses for OA and 67 common chronic conditions. We included individuals aged 40+ years on 31 December 2007, with incident OA between 2008 and 2009. We selected references without OA, matched on birth year, sex, and year of death or moving outside the region. We employed group-based trajectory modelling to capture morbidity count trajectories from 1998 to 2019. Individuals without any comorbidity were included as a reference group but were not included in the model.

**Results:**

We identified 9846 OA cases (mean age: 65.9 (SD 11.7), female: 58%) and 9846 matched references. Among both cases and references, 1296 individuals did not develop chronic conditions (no-chronic-condition class). We identified four classes. At the study outset, all classes exhibited a low average number of chronic conditions (≤1). Class 1 had the slowest progression towards multimorbidity, which increased progressively in each class. Class 1 had the lowest count of chronic conditions at the end of the follow-up (mean: 2.9 (SD 1.7)), while class 4 had the highest (9.6 (2.6)). The presence of OA was associated with a 1.29 (1.12, 1.48) adjusted relative risk of belonging to class 1 up to 2.45 (2.12, 2.83) for class 4.

**Conclusions:**

Our findings suggest that individuals with OA face an almost threefold higher risk of developing severe multimorbidity.

**WHAT IS ALREADY KNOWN ON THIS TOPIC**

- Additional chronic conditions are present in roughly 7 out of 10 people with osteoarthritis (OA), 20% more often than in age-matched and sex-matched controls. Available evidence suggests that multimorbidity is associated with incident OA which in turn appears to increase the risk of developing additional chronic conditions.

**WHAT THIS STUDY ADDS**

- This cohort study provides new insights into the multimorbidity trajectories among individuals with incident OA and OA-free matched references over a 20-year period. It identifies four classes representing different trajectories of multimorbidity progression, with class 1 having the slowest progression and class 4 having the fastest. The study reveals that individuals with OA face up to a threefold higher risk of belonging to more severe multimorbidity trajectories compared with matched OA-free references.

**HOW THIS STUDY MIGHT AFFECT RESEARCH, PRACTICE OR POLICY**

- Our findings underscore the importance of considering OA in the broader context of multimorbidity management and healthcare planning. From a policy perspective, the study could inform the development of targeted interventions to manage and potentially slow down the progression of multimorbidity in individuals with OA.

## Background

Osteoarthritis (OA) affects more than 500 million individuals globally and is a major cause of pain and functional disability worldwide.^1^ Part of the burden associated with OA is due to the high prevalence of coexisting chronic conditions, which are present in roughly 7 out of 10 people with OA; 20% more often than in age-matched and sex-matched controls.^2^ Multimorbidity represents an immediate and future challenge for healthcare systems worldwide.^3^ It is predictive of poorer quality of life, greater functional decline and increased mortality and places a considerable burden on health services, which are typically organised around a single disease model.^4–6^

Systemic factors such as low-grade inflammation and metabolic dysregulation have been advocated as the missing link between OA and multiple conditions.^7^ Recent reports reinforced this hypothesis suggesting that multimorbidity is associated with incident OA which in turn appears to increase the risk of developing additional conditions.^8–10^ These studies provide valuable information supporting a link between OA and other conditions but fail to capture the variability of multimorbidity development over time.

Previous evidence shows that distinct multimorbidity trajectories can be identified across the lifespan suggesting that multimorbidity experiences might differ between individuals.^11 12^ Nonetheless, available studies often focus on a limited number of conditions, are limited to short follow-up time (eg, 5–10 years) or do not attempt to investigate the role of OA in the multimorbidity development.^13–15^

We hypothesise that several trajectories of multimorbidity exist and that OA would occur with similar frequency in these trajectories if no association between OA and multimorbidity existed. Thus, this study aims to (1) investigate 20-year multimorbidity trajectories in a sample of individuals with incident OA and matched reference individuals without doctor-diagnosed OA, and (2) estimate the association between OA and the identified comorbidity trajectories.

## Methods

### Data

We used prospectively collected healthcare data for the population of Skåne, the southernmost region in Sweden with ~1.4 million inhabitants (one-eighth of the Swedish population in the year 2019). From the Swedish Population Register, we retrieved data on age, sex and residential addresses. Individual-level data on income, education, marital status and country of birth were retrieved from the Longitudinal Integration Database for Health Insurance and Labour Market Studies (LISA by Swedish acronym). Information on the date of death was obtained from the Swedish Cause of Death Register. Lastly, from the Skåne Healthcare Register (SHR), we extracted information about diagnoses set during any physical healthcare visit. SHR is a regional mandatory register that contains the diagnostic codes from primary and secondary care according to the International Classification of Diseases 10th Revision (ICD-10) system. These codes are assigned at the time of the healthcare visit by the physicians themselves and are automatically transferred to the register from the electronic medical records. The positive predictive value of a knee OA diagnosis in SHR has previously been reported to be high at 88%.^16^ All data from the different registers were linked through the coded personal unique identification number that is assigned to all residents in Sweden by the Swedish Tax Agency. The study is reported according to the Reporting of Studies Conducted Using Observational Routinely Collected Health Data statement.^17^

### Study design

**Figure 1:**
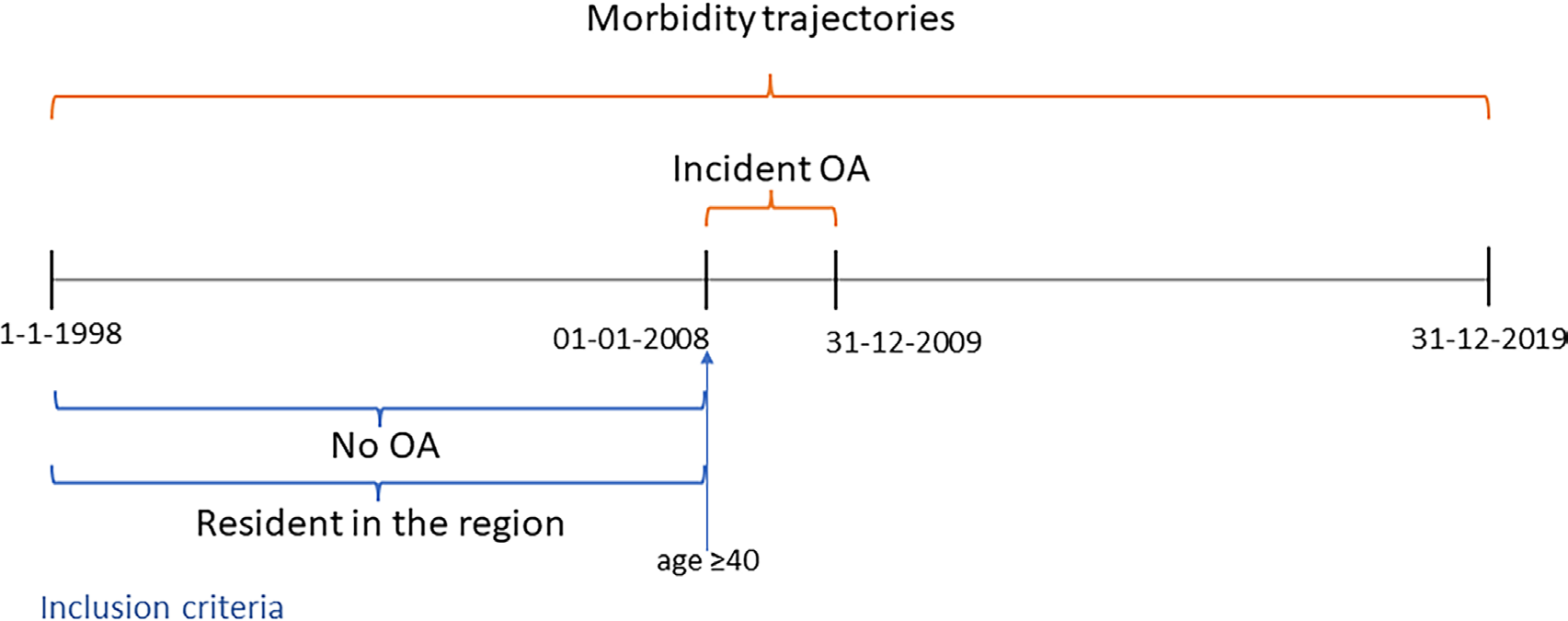
Study timeline. OA, osteoarthritis.

We performed a cohort study with incident OA used to identify cases (figure 1). We included all the individuals resident in the Skåne region aged 40 years or older on 31 December 2007, who were residing in the region from 1 January 1998 and had at least one healthcare visit (any type, not only doctor) during this time. We excluded those with a peripheral OA diagnosis (ICD-10 codes; knee: M16; hip: M17; hand: M151–M152, M18, M190D, M191D, M192D; other OA including foot, ankle, shoulder, elbow and generalised OA: all remaining M15, M18 and M19 codes) set between 1 January 1998 and 31 December 2007. Individuals were excluded even if OA was not reported as the reason for the consultation (ie, we also excluded individuals where OA was not the main diagnosis but was included as a secondary diagnosis). Among the remaining individuals without OA, we defined as cases those with an incident OA main diagnosis between 1 January 2008 and 31 December 2009 (same ICD-10 codes as above). These dates were selected to maximise the follow-up time while guaranteeing enough incident OA cases. We then matched each incident OA case to up to two reference individuals free from OA (any diagnosis, not only main) over the entire study period by birth year, sex and year of dropout (ie, death or moving from Skåne). We selected two references for each case to increase the precision of the estimates. All individuals, both cases and their respective reference, were under observation for the same time and were followed until death, relocation outside the region or 2019, whichever occurred first.

### Patient and public involvement

Patient research partners were not directly involved in the design or conduction of the study.

### Definitions and extraction of conditions

A large set of conditions were selected from a previous systematic review and previous studies from Sweden and England investigating the association between OA and incident comorbidities.^2 8 10^ In total, we included 67 conditions (online supplemental table 1a–h). We calculated the cumulative number of conditions from 1998 onwards for each OA case and reference subject. OA was excluded from the count as it was used to identify the cases.

10.1136/rmdopen-2024-004164.supp1 Supplementary data

### Disability weighting

To better reflect the disability burden carried by each condition, we decided to apply a disability weighting to the selected conditions. Disability weights represent the magnitude of health loss linked to a specific condition. For this study, we decided to use the disability weighting applied in the Global Burden of Disease (GBD) study.^18^ In this study, we computed the average disease-specific disability weight based on the disease severity distribution, which was retrieved from the GBD studies.^19–22^ The average disease-specific disability weight was calculated as the sum of the product of the multiplication between the proportion of cases in each severity state by their corresponding disability weight. As the individuals are all healthcare users, we assumed that none would be asymptomatic and we scaled the proportion of the symptomatic severity states so that their sum would be equal to 1 (online supplemental table 1a–h). Similar to the count, cumulative disability weight did not include OA which was used to identify the cases.

### Analysis

We used growth-based trajectory modelling (GBTM) to capture the variability in the trajectory of the cumulative number of morbidity counts over 20 years (for the cases, this equals 10–11 years before OA incidence and 10–11 years after).^23^ GBTM is a semiparametric, data-driven technique that divides the sample under study into subgroups (classes) of individuals that follow a similar trajectory of an outcome over time. This approach assumes no individual-level random variation within each class implying that individuals within each class follow the same trajectory. In the model, both cases and references were included together, regardless of their matched status to allow for the possibility of classifying them into different trajectories. Individuals who developed no comorbidity during the study time were not included in the model to identify trajectories and were a priori considered to belong to a separate ‘no-chronic-conditions’ trajectory. The optimal number of trajectory classes and trajectory shapes (linear, quadratic and cubic polynomials) was selected based on five criteria: (1) the lower (absolute) value of Bayesian information criteria, (2) an average posterior probability of class membership >0.7 for all classes, (3) the odds of correct classification >5 for all classes, (4) the smallest class includes at least 5% of the sample and (5) relative entropy statistics (it ranges from 0 to 1 with values closer to 1 showing a better class separation).^24^ We also accounted for model parsimony and interpretability in selecting the optimal model. After estimating the selected model, the posterior probability of membership to each trajectory class was computed and individuals were assigned to the class with the highest probability. After identifying trajectory classes, we used multinomial logistic regression to explore the association between OA and the identified trajectories, first unadjusted (model 1) and then adjusted for age and sex (model 2), and finally adjusted for sex, age, education, household individualised disposable income, marital status and being born in Sweden (model 3). We reported the estimates for all the models to allow assessment of the impact of different sets of covariates. For each of the classes, we described the prevalence of OA (which by design was present only in cases and diagnosed between 2008 and 2009), the prevalence of the other conditions, the cumulative average disability weight and mortality at the end of follow-up.

## Results

We identified 9846 persons who fulfilled our inclusion criteria and received a first diagnosis of OA between 2008 and 2009 (age mean and SD: 65.9 (11.7), female: 58.2%) (table 1). Of these, 5318 had received a first-time diagnosis of knee OA, 2479 of hip OA, 988 of hand OA, 714 of other OA and 499 of generalised OA. We also identified 19 692 reference individuals free of OA. Among the full sample (both cases and references), 1296 individuals did not develop any chronic condition (mean age: 55.2 (SD 9.0), females: 672 (51.9%), university education: 477 (36.8%)) and were grouped in a separate no-chronic-condition class which was not included in the trajectory analysis.

**Table 1:** Demographics and sample characteristics

	OA cases n=9846	References n=19 692	Total n=29 538
Age, mean (SD)	65.9 (11.7)	65.9 (11.7)	65.9 (11.7)
Female, n (%)	5734 (58.2)	11 468 (58.2)	17 202 (58.2)
Years of schooling, n (%)			
0–9	3406 (34.6)	6786 (34.5)	10 192 (34.5)
10–12	4186 (42.5)	7788 (39.5)	11 974 (40.5)
13+	2170 (22.0)	4907 (24.9)	7077 (24.0)
Missing	84 (0.9)	211 (1.1)	295 (1.0)
Marital status, n (%)			
Not married	980 (10.0)	2390 (12.1)	3370 (11.4)
Previously married	3122 (31.7)	6103 (31.0)	9225 (31.2)
Married	5744 (58.3)	11 199 (56.9)	16 943 (57.4)
Born in Sweden, n (%)	8204 (83.3)	16 361 (83.1)	24 565 (83.2)
Knee OA, n (%)	5318 (54.0)	0 (0.0)	5318 (18.0)
Hip OA, n (%)	2479 (25.2)	0 (0.0)	2479 (8.4)
Hand OA, n (%)	988 (10.0)	0 (0.0)	988 (3.3)
Other OA, n (%)	714 (7.3)	0 (0.0)	714 (2.4)
Generalised OA, n (%)	499 (5.1)	0 (0.0)	499 (1.7)

OA, osteoarthritis.

**Figure 2:**
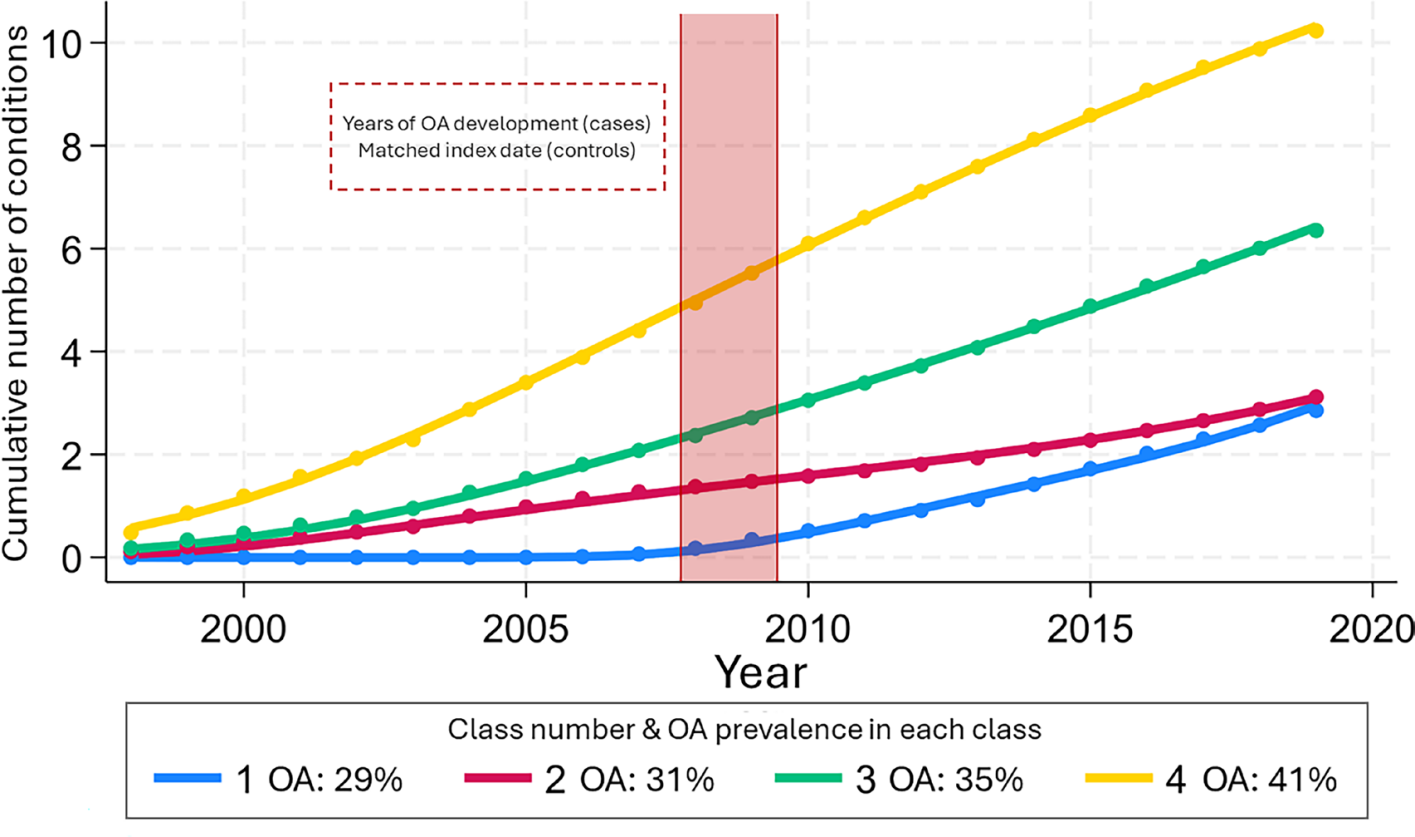
Trajectories of multimorbidity over 20 years in individuals with osteoarthritis (OA) and matched controls.

Based on the prespecified criteria, the model with four classes and a cubic trajectory shape was identified as the best fitting and most clinically relevant (online supplemental table 2). We named the classes mild multimorbidity late progression (class 1), mild multimorbidity early progression (class 2), moderate multimorbidity (class 3) and severe multimorbidity (class 4). At the start of the study period, all the classes showed a low average number of chronic conditions (≤1) (figure 2). Overall, individuals in the less severe multimorbidity classes were younger and attained higher educational levels (table 2).

**Table 2:** The baseline (index year) characteristics of the sample by trajectory class membership

	No chronic condition*	Class 1 Mild multimorbidity late progression	Class 2 Mild multimorbidity early progression	Class 3 Moderate multimorbidity	Class 4 Severe multimorbidity
Individuals, n	1296	7675	6345	9148	5074
Female, n (%)	672 (51.9)	4290 (55.9)	3748 (59.1)	5552 (60.7)	2940 (57.9)
Age, mean (SD)	55.2 (9.0)	61.8 (10.2)	62.7 (11.0)	69.0 (11.0)	73.2 (10.4)
Osteoarthritis, n (%)	323 (24.9)	2245 (29.3)	1946 (30.7)	3238 (35.4)	2094 (41.3)
Years of schooling, n (%)					
0–9	260 (20.1)	2139 (27.9)	1872 (29.5)	3535 (38.6)	2386 (47.0)
10–12	549 (42.4)	3275 (42.7)	2623 (41.3)	3655 (40.0)	1872 (36.9)
13+	477 (36.8)	2208 (28.8)	1797 (28.3)	1845 (20.2)	750 (14.8)
Missing	10 (0.8)	53 (0.7)	53 (0.8)	113 (1.2)	66 (1.3)
Marital status, n (%)					
Not married	267 (20.6)	1008 (13.1)	827 (13.0)	877 (9.6)	391 (7.7)
Previously married	253 (19.5)	1957 (25.5)	1760 (27.7)	3208 (35.1)	2045 (40.3)
Married	776 (59.9)	4708 (61.3)	3758 (59.2)	5063 (55.4)	2638 (52.0)
Household individualised disposable income (SEK100†), mean (SD)	2380 (7322)	2112 (1776)	1958 (4243)	1829 (1722)	1690 (1598)
Born in Sweden, n (%)	1044 (80.6)	6391 (83.3)	5196 (81.9)	7617 (83.3)	4317 (85.1)
Number of conditions at the end of follow-up, mean (SD)‡	0 (0)	2.9 (1.7)	3.1 (1.2)	6.1 (1.6)	9.6 (2.6)
Cumulative disability weight, mean (SD)	0 (0)	0.345 (0.248)	0.382 (0.210)	0.741 (0.272)	1.16 (0.375)
Mortality by the end of follow-up, n (%)	64 (4.9)	1056 (13.8)	1065 (16.8)	3119 (34.1)	2903 (57.2)
Knee OA, n (%)	191 (14.7)	1363 (17.8)	1179 (18.6)	1954 (21.4)	1265 (24.9)
Hip OA, n (%)	89 (6.9)	679 (8.8)	547 (8.6)	1013 (11.1)	695 (13.7)
Hand OA, n (%)	39 (3)	215 (2.8)	202 (3.2)	312 (3.4)	222 (4.4)
Other OA, n (%)	12 (0.9)	137 (1.8)	137 (2.2)	243 (2.7)	155 (3.1)
Generalised OA, n (%)	27 (2.1)	283 (3.7)	274 (4.3)	450 (4.9)	260 (5.1)

*This class was defined a priori as individuals who did not develop any chronic condition during the study time. Individuals in this class were thus not included in the model that generated classes 1–4.

†Swedish krona, 1 SEK is equivalent to ≈US$0.09 at the time of publication.

‡The count does not include OA diagnoses.

OA, osteoarthritis.

**Figure 3:**
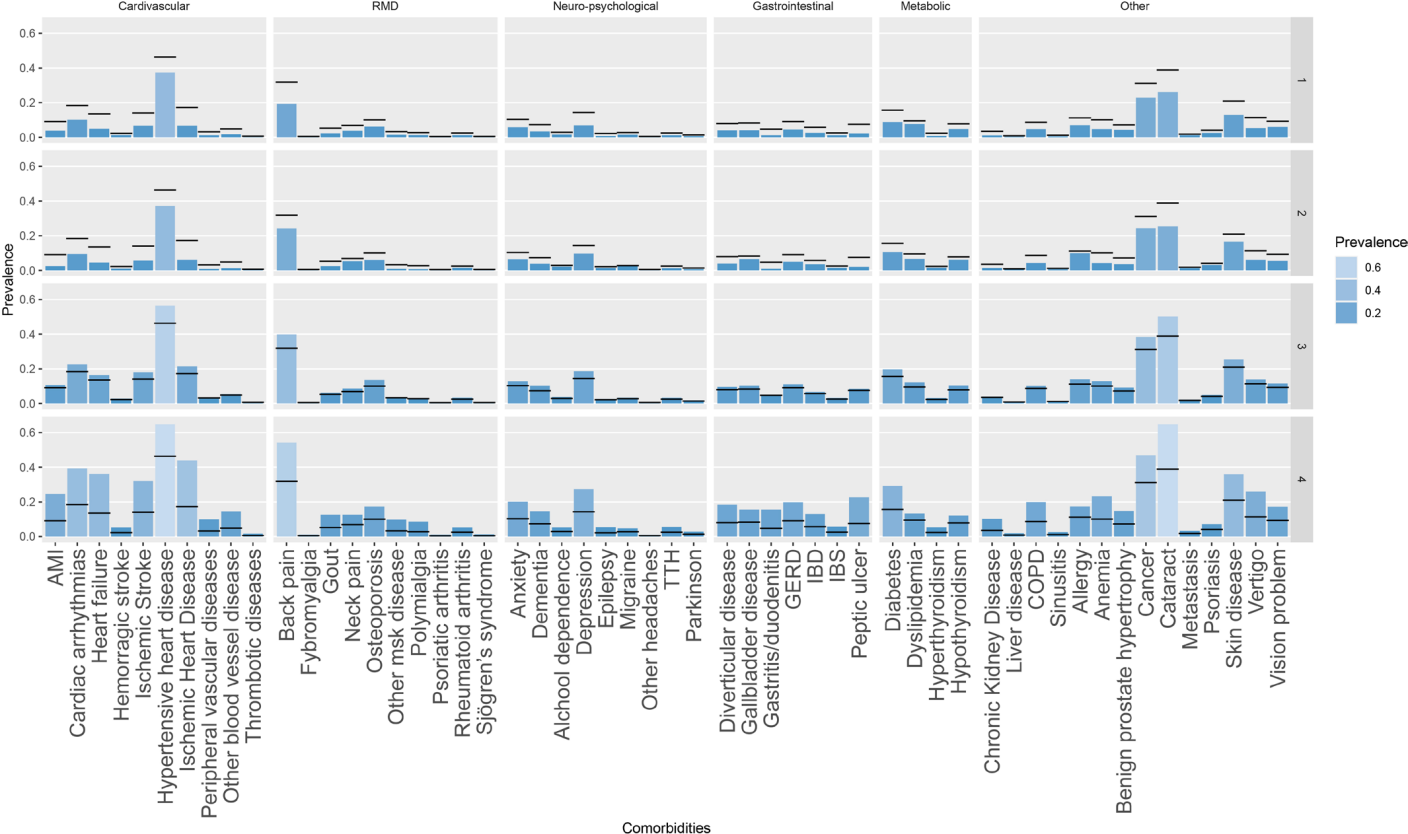
Prevalence of conditions at the end of the study period in each of the identified classes and average prevalence in the whole sample (black horizontal lines). Conditions with a prevalence lower than 1% in each of the clusters were not reported. AMI, acute myocardial infarction; COPD, chronic obstructive pulmonary disorder; GERD, gastroesophageal reflux disease; IBD, inflammatory bowel disease; IBS, irritable bowel syndrome; MSK, musculoskeletal; TTH, tension-type headache.

Progression towards multimorbidity was slowest in the mild multimorbidity classes (classes 1 and 2) and grew progressively in each class. Moreover, the mild multimorbidity late progression class showed an initial period (~10 years) with little or no development of chronic conditions, followed by a more rapid progression that brought the average number of chronic conditions in this class to approximate that of the mild multimorbidity early progression class. The count of chronic conditions at the end of follow-up was lowest in the mild multimorbidity late progression class (2.9 (SD 1.7)) and highest in the severe multimorbidity class (9.6 (2.6)) (online supplemental table 3). Moreover, mild multimorbidity classes (classes 1 and 2) consistently exhibit the lowest prevalence of any condition, while the severe multimorbidity class has the highest (figure 3, online supplemental tables 4 and 5). Disability weight and mortality followed the same pattern and were lowest in the mild multimorbidity late progression class and highest in the severe multimorbidity class, where 57% of the individuals were deceased by the end of follow-up (table 2).

The prevalence of OA was lowest in the mild multimorbidity late progression class (29.0%) and highest in the severe multimorbidity class (41.7%). The presence of OA was associated with an increased risk of assignment to any of the multimorbidity classes (classes 1–4) than no-chronic-condition class even after adjustment for age, sex, education, marital status, income and being born in Sweden. Having OA was associated with a relative risk of 1.29 (1.12, 1.48) to 2.45 (2.12, 2.83) for assignment to mild multimorbidity late progression class and severe multimorbidity class, respectively, than no-chronic-condition class (table 3). Nonetheless, the presence of OA alone showed a poor predictive value for class membership (McFadden pseudo-R^2^: 0.3).

**Table 3:** Relative risk ratio and 95% CIs for the association between osteoarthritis and comorbidity trajectory class membership when using the no-chronic-condition class as reference

	Class 1 Mild multimorbidity late progression	Class 2 Mild multimorbidity early progression	Class 3 Moderate multimorbidity	Class 4 Severe multimorbidity	McFadden pseudo-R^2^ (%)
Model 1	1.25 (1.09, 1.43)	1.33 (1.16, 1.53)	1.65 (1.44, 1.89)	2.12 (1.84, 2.43)	0.3
Model 2	1.32 (1.15, 1.51)	1.42 (1.24, 1.63)	1.87 (1.63, 2.14)	2.49 (2.15, 2.88)	6.9
Model 3	1.29 (1.12, 1.48)	1.40 (1.22, 1.61)	1.83 (1.60, 2.10)	2.45 (2.12, 2.83)	7.4

Model 1: unadjusted. Model 2: adjusted for age and sex. Model 3: adjusted for sex, age, education, income, marital status and being born in Sweden.

## Discussion

We identified four trajectories of morbidity describing progressively increasing trends in the number of chronic conditions developed over 20 years. Further, our results highlight how individuals with OA are more likely to experience more severe multimorbidity than those who do not develop OA, as they face an almost threefold higher risk of belonging to a severe multimorbidity trajectory.

Despite starting all from a low count of chronic conditions, individuals progress following very different multimorbidity trajectories. OA is more prevalent in the most severe trajectory (class 4). While this study did not aim to study the timing of OA diagnosis in relation to multimorbidity, our results suggest that OA can, in some cases, precede multimorbidity—as it can be observed in mild multimorbidity late progression class—while in others OA is diagnosed when multimorbidity is already established. These results suggest that OA is part of a disease continuum where OA and other chronic conditions concur to the development of more severe multimorbidity.^9 10^ In fact, OA alone showed a poor predictive value to identify an individual class membership further underlying the complexity of multimorbidity trajectories.

We found no discernible pattern of conditions in the identified trajectories; mild multimorbidity classes (classes 1 and 2) consistently exhibit the lowest prevalence of any conditions, while the severe multimorbidity class has the highest. Previous studies investigating clusters of comorbidities that identified specific groupings of chronic conditions often relied on a cross-sectional design, potentially missing the overall trend in the development of multimorbidity.^25 26^ Studies identifying multimorbidity clusters longitudinally have suggested that clusters are unstable over time—with more variability observed in longer follow-ups—and that a large proportion of individuals belong to unspecific clusters (with no discernable disease grouping) or die.^27 28^ Nonetheless, there is also evidence suggesting that certain diseases are more strongly correlated than others and tend to be diagnosed one after the other over short periods.^29^ Thus, subgroups with different morbidity patterns may exist within each of the trajectories at a specific time point.

When studying trajectories of multimorbidity, relying on the count of chronic conditions is a common methodological choice.^13^ However, the count of conditions may not always reflect the overall burden of those conditions on the individual—an individual with allergy and high cholesterol would have a significantly different disability burden than an individual with multiple sclerosis and rheumatoid arthritis despite the same disease count. Our results suggest that the cumulative disability weights are higher in trajectories with a higher count of conditions, thus indicating that the count of conditions can somewhat reflect the overall disability burden on the individual. In our study, the severe multimorbidity trajectory showed the highest disability burden suggesting that individuals with OA—who are at a higher relative risk of belonging to this trajectory—are more likely to experience a higher level of disability than those without OA.

While research focusing on the link between OA and multimorbidity trajectories is scarce, previous studies have used similar methods to identify multimorbidity clusters in comparable populations (older adults without OA). Despite differences in the length of the study period, studied conditions and data sources making comparisons difficult, some findings appear to be recurrent. The accumulation of chronic conditions tends most often to increase over time, rather than plateauing at a certain point, even in studies allowing for chronic conditions to be cured over time.^11 13^ In our study, all identified trajectories had the accumulation of conditions progressing over time, further strengthening the hypothesis that the accumulation of chronic conditions may represent the norm rather than the exception. Only one trajectory presented a prolonged period where the number of chronic conditions was stable. However, this happened in the first 10 years of the study period when the average count of conditions in the class was 0 (or nearly 0). Another recurrent finding is the presence of individuals who do not develop multimorbidity over time.^11 30^ We also identified a priori a class of individuals who did not develop chronic conditions during the study period. This was the smallest of the identified classes and had the lowest prevalence of OA.

Age is an important factor in multimorbidity as it increases the risk of developing chronic conditions and may even influence the rate at which conditions occur.^29^ In our study, age (which was measured at index date) was lowest in the no multimorbidity trajectory and grew progressively in each of the other trajectories confirming its strong link with multimorbidity. Our analyses show that age is an important factor in explaining multimorbidity as evidenced by a substantial increase in the model’s explained variance when age and sex were included. Nonetheless, the correlation between OA and multimorbidity remained unaffected, indicating that OA’s association with multimorbidity extends beyond age. Low physical activity, high-calorie diet and low-grade inflammation have all been advocated as a plausible link between OA and other chronic diseases and could partially explain the observed associations.^31 32^ Finally, genetic mechanisms may also play a role in the association between OA and multimorbidity.^33^

There are several limitations that need to be acknowledged. We used electronic health records to establish diseases, and thus a certain degree of misclassification can be expected (eg, individuals with OA may receive a more general code like M79 (myalgia)). Moreover, individuals who do not seek care or who seek care abroad would be missed. Due to the matched cohort design, we cannot draw any conclusion on the prevalence of the observed trajectories in the general population. Nonetheless, the estimated risks of belonging to a more severe multimorbidity trajectory can instead be generalised to individuals receiving a diagnosis of OA. Finally, we could not study the role of physical activity, diet and body weight in the relationship between OA and multimorbidity trajectories.

## Conclusions

Our findings strongly indicate that the individuals with OA are more prone to experience multimorbidity as they face an almost threefold higher risk of developing severe multimorbidity over 20 years, even after adjusting for common confounders like age, sex and socioeconomic status.

## Data Availability

Data are available upon reasonable request. All the data used in the study are available upon request to the appropriate Swedish national authorities.
