# Supplementary material for: Twenty-year trajectories of morbidity in individuals with and without osteoarthritis
Source: RMD Open. 2024 Jul 2;10(2):e004164. doi: 10.1136/rmdopen-2024-004164 (PMC11256023; doi:10.1136/rmdopen-2024-004164)
Supplement: Supplementary data [file rmdopen-2024-004164supp001.pdf]

Supplementary file

20 year trajectories of morbidity in people with and without osteoarthritis

Andrea Dell'Isola PhD[1], Filippo Recenti[1, 2], Martin Englund PhD[1], Ali Kiadaliri PhD[1]

[1]Clinical Epidemiology Unit, Orthopedics, Department of Clinical Sciences Lund, Lund University, Lund, Sweden.

[2]Department of Neurosciences, Rehabilitation, Ophthalmology, Genetics, Maternal and Child Health, University of Genova, Campus of Savona, Italy.

Corresponding author address:
Andrea Dell'Isola, Research fellow (PT, PhD)
Clinical Epidemiology Unit, Orthopedics
Department of Clinical Sciences Lund, Lund University
Wigerthuset, Remissgatan 4, Lund 22185, Sweden
andrea.dellisola@med.lu.se

Dell'Isola A, *et al. RMD Open* 2024; 10:e004164. doi: 10.1136/rmdopen-2024-004164

Supplementary table 1a: Cardiac conditions diagnostic codes and disability weights

Condition	ICD10 codes	Sequela/Severity level	Disability weight	Mean prevalence of severity level	Computed disability weight
Cardiac arrhythmias[4, 5]	I47, I48, I49	-	0.224	1	0.224
Acute myocardial infarction (AMI) [4, 5]	I21-24	First 2 days	0.432*	0.07	0.108
		2-28 days	0.074*	0.93	
Ischemic heart disease[3*]	I20, I25	Mild	0.033	0.33	0.108
		Moderate	0.08	0.18	
		Severe	0.167	0.50	
Heart failure[5]	I50, I34-I37	Mild	0.041	0.68	0.073
		Moderate	0.072	0.12	
		Severe	0.179	0.20	
Hypertensive heart disease[4, 5]	I10, I11, I12, I13, I15	General severity for chronic disease	0.049	1	0.049
Peripheral vascular diseases (claudication, Raynaud syndrome, Buerger's disease) [5]	I73	-	0.014	1	0.014
Other blood vessel diseases (atherosclerosis and aneurysm) [5]	I70, I71, I72	-	0.014	1	0.014
Thrombotic diseases[5]	I74	-	0.014	1	0.014
Ischemic Stroke[2]	G45, G46, I63, I65, I66, I69.3, I69.4, I69.8	Mild	0.019	0.526	0.104
		Moderate	0.07	0.279	
		Moderate + cognition problems	0.316	0.144	
		Severe	0.552	0.020	
		Severe + cognition problems	0.588	0.031	
Hemorrhagic stroke[2]	I61-I62, I69.0-I69.2 I67.0-I67.1		0.019	0.43	0.161

* the weights are based on prevalence of angina I20

Supplementary table 1b: Rheumatic and musculoskeletal conditions diagnostic codes and disability weights

Condition	ICD10 code	Sequela/Severity level	Disability weight	Mean prevalence of severity level	Computed disability weight
Back pain[4]	M40, M47.1-M47.2, M48*, M51.0-M51.2, M53.2-.9, M54.1 M54.3-9, *, M99.0, m99.1	Mild without leg pain	0.02	0.41	0.122
		Moderate without leg pain	0.054	0.35	
		Severe without leg pain	0.272	0.10	
		Most severe without leg pain	0.372	0.14	
		Mild without leg pain	0.02	0.27	
		Moderate with leg pain	0.054	0.36	
		Severe with leg pain	0.325	0.14	
		Most severe with leg pain	0.384	0.23	
Neck pain[4]	G54.2, M50, M51.3-M51.9*, M53.0, 53.1, M54.2, M99.1A	Mild	0.052	0.67	0.107
		Moderate	0.112	0.12	
		Severe	0.226	0.06	
		Most severe	0.300	0.15	
Rheumatoid arthritis [3]	M05, M06	Mild	0.117	0.47	0.261
		Moderate	0.317	0.39	
		Severe	0.581	0.14	
Gout [3]	M10	Episodes of gout	0.295	0.87	0.332
		Poliarticular gout	0.581	0.13	
Sjögren's syndrome *	M35.0	Musculoskeletal problems, lower limbs, mild	0.023	0.306	0.130
		Musculoskeletal problems, upper limbs, mild	0.028	0.278	
		Musculoskeletal problems, upper limbs, moderate	0.115	0.139	
		Musculoskeletal problems, lower limbs, severe	0.163	0.083	
		Musculoskeletal problems, generalised, moderate	0.312	0.097	
		Musculoskeletal problems, generalised, severe	0.572	0.097	
Systemic lupus erythematosus (SLE) *	M32				0.130

Other msk disease (other inflammatory diseases) *	M35.1-M35.9		0.130
Ankylosing spondylitis*	M45		0.130
Psoriatic arthritis*	M07		0.130
Polymyalgia rheumatica*	M353		0.130
Fibromyalgia*	M79.7		0.130
Osteoporosis [5]	M80-M82		0.131

*General weights for RMD conditions not elsewhere defined. Calculation is reported only for Sjögren's syndrome

Supplementary table 1c: Neuropsychological conditions diagnostic codes and disability weights

Condition	ICD10 code	Sequela/Severity level	Disability weight	Mean prevalence of severity level	Final computed DW
Dementia[4] <70*	F00, F01, F02, F03, G30, G31.0 - G31.1, G31.8 – G31.9, G32.8	Mild	0.069	0.79	0.138
		Moderate	0.377	0.15	
		Severe	0.449	0.06	
Dementia[4] 70-79*		Mild	0.069	0.77	0.145
		Moderate	0.377	0.16	
		Severe	0.449	0.07	
Dementia[4] 80+*		Mild	0.069	0.68	0.174
		Moderate	0.377	0.23	
		Severe	0.449	0.09	
Depression[4]	F32, F33	Mild	0.145	0.686	0.254
		Moderate	0.396	0.198	
		Severe	0.658	0.116	
Anxiety[4, 5]	F41	Mild	0.03	0.55	0.146
		Moderate	0.133	0.27	
		Severe	0.523	0.18	
Eating disorders (Anorexia / Bulimia nervosa) [4]	F50	-	0.224	1	0.224
Epilepsy[3]	G40, G41	Less severe (seizures < once per Month)	0.263	0.48	0.413
		Severe (seizures >= once per Month)	0.552	0.52	
TTH[3 5]	G44.2	-	0.037	0.029[#]	0.001
Migraine [3, 5]	G43	-	0.441	0.093[#]	0.041

4

Medication overuse [3, 5]	G44.4	-	0.223	0.532[#]	0.119
Other headaches	G44.0, G44.1, G44.3, G44.8	General severity for chronic disease			0.049
Multiple Sclerosis[3]	G35	Mild	0.183	0.49	0.366
		Moderate	0.463	0.36	
		Severe	0.719	0.15	
Parkinson disease[3]	G20	Mild	0.01	0.21	0.278
		Moderate	0.267	0.58	
		Severe	0.575	0.21	
Schizophrenia[3]	F20, F25	Schizophrenia residual	0.588	0.63	0.658
		Schizophrenia acute state	0.778	0.37	
Alcohol dependence[3]	F10	Mild	0.123	0.79	0.181
		Moderate	0.235	0.07	
		Severe	0.373	0.06	
		Most Severe	0.57	0.08	
Opioid dependence[3]	F11	Mild	0.335	0.44	0.538
		Moderate-severe	0.697	0.56	
Cocaine dependence[3]	F14	Mild	0.116	0.5	0.2975
		Moderate-severe	0.479	0.5	
Cannabis dependence[3]	F12	Mild	0.039	0.86	0.07
		Moderate-severe	0.266	0.14	
Other dependence[3]	F15	Mild	0.079	0.42	0.314
		Moderate-severe	0.486	0.58	

* The severity distribution and associated disability weights for dementia were combined to compute an average DW for the following three age groups: 0.152. [#]time spent symptomatic

Supplementary table 1d: Gastrointestinal conditions diagnostic codes and disability weights

Condition	ICD10 code	Sequela/Severity level	Disability weight	Mean prevalence of severity level	Final computed DW
Gallbladder and biliary diseases[3]	K80-K83	Mild	0.011	0.59	0.278
		Moderate	0.114	0.25	
		Severe	0.324	0.16	
GERD[5]* (oesophagus diseases)	K20, K21, K22.1, k22.3-K22.9, R12	Mild	0.027	0.72	0.051
		Moderate-severe	0.114	0.28	
Gastritis/duodenitis[3]	K29	Mild with no anemia	0.011	0.23	0.075
		Mild with mild anaemia	0.015	0.16	

		Mild with moderate anaemia	0.062	0.13	
		Mild with severe anemia	0.158	0.01	
		Moderate with no anaemia	0.114	0.20	
		Moderate with mild anaemia	0.118	0.14	
		Moderate with moderate anaemia	0.16	0.12	
		Moderate with severe anaemia	0.246	0.01	
**Peptic ulcer disease[3]**	K25-K29	Mild without anaemia	0.011	0.15	0.087
		Mild with mild anaemia	0.015	0.15	
		Mild with moderate anaemia	0.062	0.12	
		Mild with severe anaemia	0.158	0.01	
		Moderate with no anaemia	0.114	0.20	
		Moderate with mild anaemia	0.118	0.20	
		Moderate with moderate anaemia	0.16	0.17	
		Moderate with severe anaemia	0.246	0.01	
**Inflammatory bowel disease (IBD) [3]**	K50, K51, K52	-	0.231	1	0.231
**Irritable bowel syndrome (IBS) [3]**	K58	General severity for chronic disease	0.049	1	0.049
**Diverticular disease of intestine[3]**	K57	General severity for chronic disease	0.049	1	0.049

Supplementary table 1e: liver and kidney conditions diagnostic codes and disability weights

Condition	ICD10 code	Sequela/Severity level	Disability weight	Mean prevalence of severity level	Final computed DW
Cirrhosis	K70-k74	-	0.181	1	0.181
Chronic Kidney Disease (CKD) [6]	N02, N03, N04, N05, N06.0, N07, N08, N11, N12.9, N15, N16, N18, N19	Stage 3	0.068	0.94	0.078
		Stage 4	0.150	0.05	
		Stage 5	0.590	0.01	

Supplementary table 1f: metabolic conditions diagnostic codes and disability weights

Condition	ICD10 code	Sequela/Severity level	Disability weight	Mean prevalence of severity level	Final computed DW
Diabetes mellitus[5]	E10.0 , E11, E12; E13, E14	-		1	0.077
Dyslipidaemia [3]	E78	General severity for chronic disease		1	0.049
Hyperthyroidism[3]	E05	General severity for chronic disease		1	0.049
Hypothyroidism[3]	E03	General severity for chronic disease		1	0.049

Supplementary table 1g: respiratory conditions diagnostic codes and disability weights

Condition	ICD10 code	Sequela/Severity level	Disability weight	Mean prevalence of severity level	Final computed DW
Chronic obstructive pulmonary disease (COPD) [3]	J41, J43, j42.9, J44	Mild	0.019	0.64	0.13
		Moderate	0.225	0.16	
		Severe	0.408	0.20	
Chronic sinusitis	J32	General severity for chronic disease		1	0.049

Dell'Isola A, *et al. RMD Open* 2024; 10:e004164. doi: 10.1136/rmdopen-2024-004164

Supplementary table 1h: other conditions diagnostic codes and disability weights

Condition	ICD10 code	Sequela/Severity level	Disability weight	Mean prevalence of severity level	Final computed DW
Benign prostate hypertrophy (BPH) [5]	N40.9	-			0.067
Anaemia (all types) [5]	D50 – D64	-			0.028
Eczema/Skin disease	L20-L30	General severity for chronic disease			0.049
Cancer*	C00-C43, C45-C78, C81-C97	Primary neoplasm: diagnosis and primary therapy	0.288		0.288
Metastasis	C79-C80	Secondary Neoplasm (metastasis)	0.451		0.451
Psoriasis	L40, L41	Mild	0.027	0.79	0.094
		Mod	0.188	0.12	
		Severe	0.576	0.09	
Severe allergy	J30, T78 Z88	General severity for chronic disease			0.049
Vertigo	R42.9, H81.4	-			0.113
Vision problem (glaucoma and other) [3]	H27, H40, H42	Moderate	0.03	0.10	0.170
		Severe	0.184	0.49	
		Blindness	0.187	0.41	
Cataract[3]	H25, H26, H28	Moderate	0.03	0.69	0.078
		Severe	0.184	0.16	
		Blindness	0.187	0.14	
Sleep (insomnia)	F51.0	General severity for chronic disease			0.049

Supplementary Table 2: Models fit

# of classes	BIC	Relative entropy	%class 1	%class 2	%class 3	%class 4	%class 5	%class 6	%class 7	%class 8	Average posterior probability of assignment (APPA)	Odds of correct classification (OCC)
2	923900	0.965	63.2	36.8							0.992/0.985	76/115
3	850327	0.961	39.7	43.5	16.8						0.985/0.980/0.983	98/63/281
4	806107	0.960	25.1	38.8	26.9	9.2					0.982/0.975/0.974/0.984	163/61/100/603
5	781767	0.951	21.4	28.8	27.4	16.8	5.6				0.977/0.961/0.958/0.973/0.985	156/61/60/176/1112
6	758348	0.952	21.7	22.2	18.6	16.3	5.5	15.7			0.982/0.963/0.951/0.948/0.985 /0.970	198/90/85/93/1159/ 172
7	741424	0.953	19.9	18.8	16.9	11.5	18.0	11.2	3.6		0.982/0.957/0.951/0.946/0.955 /0.972/0.986	230/95/96/133/97/276 /1845
8	726555	0.952	11.4	19.2	17.0	15.0	9.6	9.4	15.3	3.0	0.976/0.957/0.960/0.945/0.946 /0.978/0.953/0.986	320/94/117/97/164/ 418/113/2305

42 Supplementary Table 3: prevalence of final cumulative count of chronic conditions in each class

	No chronic condition*	Class 1, Mild multimorbidity late progression	Class 2, Mild multimorbidity early progression	Class 3, Moderate multimorbidity	Class 4, Severe multimorbidity
Number of individuals	1,296	7,675	6,345	9,148	5,074
Number of conditions at the end of follow-up, n (%)					
**0**	1296 (100)	0 (0)	0 (0)	0 (0)	0 (0)
**1**	0 (0)	1960 (25.5)	567 (8.9)	0 (0)	0 (0)
**2**	0 (0)	1811 (23.6)	1476 (23.3)	49 (0.5)	0 (0)
**3**	0 (0)	1500 (19.5)	2119 (33.4)	310 (3.4)	3 (0.1)
**4**	0 (0)	1062 (13.8)	1483 (23.4)	1146 (12.5)	44 (0.9)
**5**	0 (0)	735 (9.6)	570 (9)	2044 (22.3)	128 (2.5)
**6**	0 (0)	344 (4.5)	110 (1.7)	2195 (24)	309 (6.1)
**7**	0 (0)	182 (2.4)	18 (0.3)	1677 (18.3)	559 (11)
**8**	0 (0)	59 (0.8)	2 (0)	1013 (11.1)	802 (15.8)
**9**	0 (0)	16 (0.2)	0 (0)	486 (5.3)	848 (16.7)
**10**	0 (0)	4 (0.1)	0 (0)	173 (1.9)	748 (14.7)
**11**	0 (0)	1 (0)	0 (0)	43 (0.5)	592 (11.7)
**12**	0 (0)	1 (0)	0 (0)	11 (0.1)	396 (7.8)
**13**	0 (0)	0 (0)	0 (0)	1 (0)	286 (5.6)
**14**	0 (0)	0 (0)	0 (0)	0 (0)	149 (2.9)
**15**	0 (0)	0 (0)	0 (0)	0 (0)	87 (1.7)
**16**	0 (0)	0 (0)	0 (0)	0 (0)	53 (1)
**17**	0 (0)	0 (0)	0 (0)	0 (0)	30 (0.6)
**18**	0 (0)	0 (0)	0 (0)	0 (0)	21 (0.4)
**19**	0 (0)	0 (0)	0 (0)	0 (0)	9 (0.2)
**20**	0 (0)	0 (0)	0 (0)	0 (0)	2 (0)
**21**	0 (0)	0 (0)	0 (0)	0 (0)	6 (0.1)
**22**	0 (0)	0 (0)	0 (0)	0 (0)	1 (0)
**24**	0 (0)	0 (0)	0 (0)	0 (0)	1 (0)

* This class was defined a priori as individuals who did not develop any chronic condition during the study time. Individuals in this class were thus not included in the model that generated classes 1 to 4.

BMJ Publishing Group Limited (BMJ) disclaims all liability and responsibility arising from any reliance placed on this supplemental material which has been supplied by the author(s)

Supplementary Table 4: prevalence of major groupings of chronic conditions by class*

Conditions, n (%)	Class 1, Mild multimorbidity late progression	Class 2, Mild multimorbidity early progression	Class 3, Moderate multimorbidity	Class 4, Severe multimorbidity	Total
Cardiovascular	4,117 (54)	3,380 (53)	7,383 (81)	4,705 (93)	19,585 (66)
RMD	2,364 (31)	2,336 (37)	5,255 (57)	3,694 (73)	13,649 (46)
Neuro-psychological	1,483 (19)	1,630 (26)	3,821 (42)	2,797 (55)	9,731 (33)
Gastrointestinal	1,303 (17)	1,331 (21)	3,560 (39)	3,057 (60)	9,251 (31)
Liver and Kidney	117 (2)	123 (2)	464 (5)	619 (12)	1,323 (5)
Metabolic	1,587 (20.7)	1,460 (23.0)	3,533 (38.6)	2,474 (48.8)	9,054 (30.7)
Respiratory	418 (5.4)	317 (5.0)	1,066 (11.7)	1,110 (21.9)	2,911 (9.9)
Other	5,036 (66)	4,399 (69)	8,201 (90)	4,871 (96)	22,507 (76)

RMD: Rheumatic Musculoskeletal Disease

Supplementary Table 5: prevalence of chronic conditions by class*

Conditions, n (%)	Class									
	Class 1, Mild multimorbidity late progression		Class 2, Mild multimorbidity early progression		Class 3, Moderate multimorbidity		Class 4, Severe multimorbidity		Total	
AMI	295	(3,8)	160	(2,5)	978	(10,7)	1,249	(24,6)	2,682	(9,1)
Cardiac arrhythmias	776	(10,1)	597	(9,4)	2,06	(22,5)	1,995	(39,3)	5,428	(18,4)
Heart failure	376	(4,9)	284	(4,5)	1,502	(16,4)	1,829	(36,0)	3,991	(13,5)
Hemorrhagic stroke	94	(1,2)	62	(1,0)	259	(2,8)	264	(5,2)	679	(2,3)
Ischemic Stroke	520	(6,8)	358	(5,6)	1,644	(18,0)	1,623	(32,0)	4,145	(14,0)
Hypertensive heart disease	2,864	(37,3)	2,356	(37,1)	5,168	(56,5)	3,29	(64,8)	13,678	(46,3)
Ischemic Stroke	521	(6,8)	386	(6,1)	1,963	(21,5)	2,228	(43,9)	5,098	(17,3)
Peripheral vascular diseases	90	(1,2)	51	(0,8)	309	(3,4)	507	(10,0)	957	(3,2)
Other blood vessel disease	129	(1,7)	76	(1,2)	486	(5,3)	736	(14,5)	1,427	(4,8)
Thrombotic diseases	12	(0,2)	16	(0,3)	76	(0,8)	86	(1,7)	190	(0,6)
Back pain	1,478	(19,3)	1,538	(24,2)	3,641	(39,8)	2,749	(54,2)	9,406	(31,8)
Fibromyalgia	17	(0,2)	10	(0,2)	68	(0,7)	55	(1,1)	150	(0,5)
Gout	181	(2,4)	159	(2,5)	570	(6,2)	638	(12,6)	1,548	(5,2)
Neck pain	293	(3,8)	333	(5,2)	787	(8,6)	639	(12,6)	2,052	(6,9)
Osteoporosis	472	(6,1)	378	(6,0)	1,24	(13,6)	876	(17,3)	2,966	(10,0)
Other msk disease	110	(1,4)	54	(0,9)	319	(3,5)	494	(9,7)	977	(3,3)
Polymialgia	97	(1,3)	40	(0,6)	256	(2,8)	433	(8,5)	826	(2,8)
Psoriatic arthritis	8	(0,1)	15	(0,2)	49	(0,5)	56	(1,1)	128	(0,4)
Rheumatoid arthritis	93	(1,2)	90	(1,4)	304	(3,3)	265	(5,2)	752	(2,5)
Sjögren's syndrome	12	(0,2)	19	(0,3)	50	(0,5)	64	(1,3)	145	(0,5)
Anxiety	443	(5,8)	412	(6,5)	1,171	(12,8)	1,023	(20,2)	3,049	(10,3)
Dementia	263	(3,4)	244	(3,8)	934	(10,2)	743	(14,6)	2,184	(7,4)
Alcohol dependence	120	(1,6)	144	(2,3)	345	(3,8)	268	(5,3)	877	(3,0)
Depression	534	(7,0)	612	(9,6)	1,698	(18,6)	1,384	(27,3)	4,228	(14,3)
Epilepsy	53	(0,7)	81	(1,3)	243	(2,7)	278	(5,5)	655	(2,2)
Migraine	114	(1,5)	175	(2,8)	304	(3,3)	238	(4,7)	831	(2,8)
Other headaches	21	(0,3)	24	(0,4)	55	(0,6)	51	(1,0)	151	(0,5)
TTH	87	(1,1)	75	(1,2)	303	(3,3)	285	(5,6)	750	(2,5)
Parkinson	50	(0,7)	44	(0,7)	169	(1,8)	145	(2,9)	408	(1,4)
Diverticular disease	309	(4,0)	250	(3,9)	876	(9,6)	929	(18,3)	2,364	(8,0)

Gallbladder disease	312	(4,1)	417	(6,6)	935	(10,2)	792	(15,6)	2,456	(8,3)
Gastritis/duodenitis	90	(1,2)	56	(0,9)	445	(4,9)	792	(15,6)	1,383	(4,7)
GERD	343	(4,5)	314	(4,9)	1,016	(11,1)	1,007	(19,8)	2,68	(9,1)
IBD	201	(2,6)	226	(3,6)	612	(6,7)	659	(13,0)	1,698	(5,7)
IBS	88	(1,1)	91	(1,4)	294	(3,2)	294	(5,8)	767	(2,6)
Peptic ulcer	177	(2,3)	134	(2,1)	776	(8,5)	1,147	(22,6)	2,234	(7,6)
Chronic Kidney Disease	81	(1,1)	82	(1,3)	359	(3,9)	521	(10,3)	1,043	(3,5)
Liver disease	27	(0,4)	33	(0,5)	91	(1,0)	98	(1,9)	249	(0,8)
Diabetes	676	(8,8)	666	(10,5)	1,806	(19,7)	1,484	(29,2)	4,632	(15,7)
Dyslipidemia	594	(7,7)	425	(6,7)	1,117	(12,2)	672	(13,2)	2,808	(9,5)
Hyperthyroidism	57	(0,7)	96	(1,5)	290	(3,2)	271	(5,3)	714	(2,4)
Hypothyroidism	367	(4,8)	392	(6,2)	950	(10,4)	615	(12,1)	2,324	(7,9)
COPD	361	(4,7)	269	(4,2)	919	(10,0)	1,011	(19,9)	2,56	(8,7)
Sinusitis	41	(0,5)	40	(0,6)	140	(1,5)	130	(2,6)	351	(1,2)
Allergy	540	(7,0)	627	(9,9)	1,271	(13,9)	879	(17,3)	3,317	(11,2)
Anemia	363	(4,7)	266	(4,2)	1,176	(12,9)	1,175	(23,2)	2,98	(10,1)
Benign prostate hypertrophy	330	(4,3)	230	(3,6)	837	(9,1)	751	(14,8)	2,148	(7,3)
Cancer	1,756	(22,9)	1,548	(24,4)	3,517	(38,4)	2,382	(46,9)	9,203	(31,2)
Cataract	2,006	(26,1)	1,616	(25,5)	4,585	(50,1)	3,291	(64,9)	11,498	(38,9)
Metastasis	92	(1,2)	78	(1,2)	208	(2,3)	168	(3,3)	546	(1,8)
Psoriasis	197	(2,6)	201	(3,2)	449	(4,9)	364	(7,2)	1,211	(4,1)
Skin disease	995	(13,0)	1,053	(16,6)	2,328	(25,4)	1,822	(35,9)	6,198	(21,0)
Vertigo	403	(5,3)	382	(6,0)	1,263	(13,8)	1,317	(26,0)	3,365	(11,4)
Vision problem	457	(6,0)	345	(5,4)	1,058	(11,6)	872	(17,2)	2,732	(9,2)

***** Conditions with a prevalence lower than 1% in each of the clusters were not reported, AMI: acute myocardial infarction, COPD: chronic obstructive pulmonary disorder, GERD: gastroesophageal reflux diseases, IBD: inflammatory bowel disease, IBS: irritable bowel syndrome, TTH: tension-type headache,
